# OD37 How The PICOTS-ComTeC Framework For Digital Health Interventions Can Be Useful For Health Technology Assessment

**DOI:** 10.1017/S0266462324001661

**Published:** 2025-01-07

**Authors:** Zsombor Zrubka, Annette Champion, Rossella Di Bidino, Anke-Peggy Holtorf, Jagadeswara R Earla, Artem Boltyenkov, Masami Tabata-Kelly, Carl Asche, Anita Burrell

## Abstract

**Introduction:**

The diffusion of digital health interventions (DHIs) requires agreement on a minimum information framework to define them. The ISPOR Digital Health (DH) Special Interest Group (SIG) developed the PICOTS-ComTeC framework based upon a systematic review and a Delphi study. It is an enriched version of the traditional PICOTS widely adopted in health technology assessment (HTA). ComTeC stands for communication, technology, and context.

**Methods:**

The PICOTS-ComTeC is based upon a review that included the Shannon–Weaver model of communication, AHRQ Quality Measures, technology, geography, and the World Health Organization classification of DHIs followed by a Delphi panel. The development process adhered to the EQUATOR guidelines. The PICOTS-ComTeC aims to be a flexible and versatile tool tested on different DHIs. The results of the testing are discussed from the HTA perspective considering the tool’s additional value, utility for and applicability to HTA. The additional value is strictly linked to the actual need for a dedicated PICOTS for DHIs and its implications for HTA assessments of DHIs.

**Results:**

The PICOTS-ComTeC was tested internally and externally to the ISPOR DH-SIG on four DHIs for breast cancer surgery/management/patient education, one DHI for obesity, and one DHI for patients with heart failure. The testing phase demonstrated the level of detail required to use the tool, hitherto available evidence to cover all domains, and opened up discussion on implications of the PICOTS-ComTeC framework for HTA related activities (i.e., scoping, literature search, comparator selection). It emerged that there is a diffuse lack of homogeneity and details when DHIs are defined in the literature with significant implications for conducting appropriate HTAs.

**Conclusions:**

The diffuse adoption of the PICOTS-ComTeC for patient-facing DHIs will promote a greater level of detail in order to define homogenous DHI groups. The implications for HTA range from the definition of relevant research questions to the selection of the most appropriate comparator so that assessments are geared to fulfill the needs of decision-makers.

